# Red Deer (*Cervus elaphus*) Fascioloidosis: From Liver Pathology to Regeneration

**DOI:** 10.3390/life16030502

**Published:** 2026-03-19

**Authors:** Dean Konjević, Nikolina Škvorc, Miljenko Bujanić, Jan Čurlík, Anđelko Gašpar, Ivan-Conrado Šoštarić-Zuckermann, Andrea Gudan Kurilj

**Affiliations:** 1 Faculty of Veterinary Medicine, University of Zagreb, Heinzelova 55, 10000 Zagreb, Croatia; nskvorc@vef.unizg.hr (N.Š.); mbujanic@vef.unizg.hr (M.B.); isostaric@vef.unizg.hr (I.-C.Š.-Z.); agudan@vef.unizg.hr (A.G.K.); 2 Department of Breeding and Diseases of Game, Fish and Bees, Ecology and Cynology, University of Veterinary Medicine and Pharmacy Košice, Komenského 73, 041 81 Košice, Slovakia; jan.curlik@uvlf.sk; 3 Croatian Veterinary Chamber, Heinzelova 55, 10000 Zagreb, Croatia; hvk@hvk.hr

**Keywords:** *Fascioloides magna*, red deer, liver injury, regeneration

## Abstract

Fascioloidosis is a parasitic disease caused by allochthonous parasite *Fascioloides magna*. In Europe, three types of final hosts are recognised: definitive, aberrant, and dead end. Several countries have launched disease control programmes using medicated feed, with different drugs, to control *F. magna* infections. In this study, we used corn treated with Albix^®^ 10 in a total dose of 60 mg/kg of body weight for five consecutive days (12 mg/kg per day). Following successful treatment, a destroyed pseudocyst with different amounts of degrading material and decaying flukes was detected. A total of 136 livers was examined. The average number of pseudocysts per positive liver was seven (min. 1–max. 45), while the average number of adult flukes was 14.17 (2–70). On average, 1.34 juvenile flukes in the migratory phase were detected per infected liver. The average number of pseudocysts was 7.07 per liver in total. Degrading pseudocysts were either absent or present to a maximum of 120 per liver, with an average of 7.99 per liver. Some livers had multifocal to confluent nodules bulging from the liver parenchyma, which were up to 7 cm in diameter. Histologically, these areas showed disruption, containing bands of fibrous connective tissue, dividing parenchyma into pseudolobules of varying size and shape. These septa contained dark brown to black pigment (iron porphyrin), along with remnants of elliptical, operculated, mainly empty trematode eggs. Nodules were surrounded with fibrous tissue and disorganised hyperplastic hepatocytes arranged in irregular trabeculae supported by fibrous bands occasionally containing blood vessels. This study shows the potential of liver regeneration in the case of acute and chronic liver injury, as well as in cases of fatty liver disease.

## 1. Introduction

*Fascioloides magna* is a North American trematode that parasitises the liver of various domestic and wild species [1]. After ingestion of the metacercariae (infective stage encysted on the vegetation), the juvenile fluke penetrates the intestinal wall and migrates along the ventral abdominal surface toward the liver. Sometimes, especially in the case of less adequate hosts, juvenile flukes can end up in other organs such as the spleen or lungs, causing tissue damage and bacterial infections [2]. Only after entering these organs can the fluke revert its migration and continue back toward the liver, where it will begin its migration through the liver parenchyma. The fluke’s migration results in tissue damage and the formation of the migratory channels, or in the case of an aberrant hosts, it can cause excessive damage and haematoma [1]. It is thought that such migration partially enables the fluke to avoid immune responses of the host. In normal cases, when the migration is over, the host will form a pseudocyst, which is an imperfect barrier that usually encapsulates two adult trematodes. The pseudocysts can be located in any part of the liver, but are typically found in the middle part [3], which is rich in blood vessels and bile ducts. Depending on the host type and potential anthelmintic treatment, liver lesions can vary in type and severity. In the case of definitive hosts like red deer (*Cervus elaphus*) in Europe, gross lesions of the liver include surface irregularities, loss of translucency of the Glisson’s capsule, fibrin deposits, adhesions between the liver and diaphragm, traces of black pigment (iron porphyrin) visible on both the outer and cut surface, fluke migratory channels, and thin-walled pseudocysts [1]. Pseudocysts can be single, confluent, small or even as large as a human fist. It is believed that the fluke can survive for up to five years in these pseudocysts.

Following the detection of this allochthonous parasite in Europe and the establishment of three main natural foci of infection, several countries have applied anthelmintic treatment of red, fallow (*Dama dama*) and roe deer (*Capreolus capreolus*). Different drugs have been applied perorally at the population level, using corn, salt or special mixtures as carriers [4,5,6,7,8]. Successful treatment results in damage to the fluke’s tegument and consequent degradation of pseudocyst content. Degrading pseudocysts are filled with yellow amorphous liquid that occasionally contains remnants of the fluke. The actual fate of this content and its potential impact on the host’s organism, especially in the case of a high number of pseudocysts, remained unknown for long period of time. The aim of this study is to present the nature of gross and microscopic lesions in the red deer livers before and after drug consumption, starting from migration, pseudocysts with live flukes, degrading pseudocysts and, for the first time, describing the formation of regenerative areas.

## 2. Material and Methods

### 2.1. Study Location and Samples

A total of 136 red deer livers were collected during the 2024/2025 hunting season as part of the regular game management operations in the Bjelovar–Bilogora County in northwest Croatia. The area is characterised by lowland to hilly mountain areas (elevation range from 97 to 885 m) abundant in natural water streams. Forest associations are mainly represented with downy oak and narrow-leaved ash (*Orno*—*Quercetum pubescentis*), sessile oak-birch (*Betulo*–*Quercetum petrae*), beech-greater wood rush (*Luzulo*-*Fagetum*), fir-beech (*Abieti* –*Fagetum panonicum*), common hornbeam–pedunculate oak (*Carpino betuli*-*Quercetum roboris*), and others. According to the W. Köppen classification, this area belongs to the Cfb climate, with equally distributed precipitation over the year, and average temperatures not lower than −3 °C during the winter, while the highest average temperature during the summer is less than 22 °C [9]. In the late winter (early February) of 2024, following the end of the hunting season, animals were given anthelmintic treatment using albendazole (Albix^®^ 10). We use drug rotation over the seasons to avoid potential development of drug resistance. The drug was administered on corn, based on the calculated herd size (performed by game managers), average individual live body weight of 110 kg, and approximate meal size. Medicated feed was prepared for five days (each day the same amount of corn was provided) with a calculated total dose of 60 mg/kg (daily dose of 12 mg/kg) of body weight in total. Treatment was performed in accordance with instructions given by Ursprung et al. [8]. During the 2024/2025 hunting season (September 2024 to 15 February 2025), livers were collected from culled deer, stored in plastic bags and frozen until further analysis. All livers were collected as non-probability convenient sampling, thanks to the courtesy of hunters.

### 2.2. Macroscopic Analysis

The external surface of thawed livers was analysed for visible signs indicative of *F. magna* infection (surface irregularities, fibrin deposits, traces of iron porphyrin, liver enlargement). Following external inspection, each liver was cut into slices of approximately 2 cm thickness and thoroughly examined on both sides. The presence of gross lesions (fluke migratory channels, active pseudocysts, degraded pseudocysts and regeneration areas) and parasite developmental stages (juvenile, adult and degrading fluke) was analysed and noted.

### 2.3. Histological Analysis

Tissue samples were collected from each type of gross lesion, including both part of the lesion and part of the surrounding healthy tissue. Collected samples were fixed in 10% neutral buffered formalin, embedded in paraffin and sectioned to an approximate thickness of 6 μm. Prepared sections were stained using routine haematoxylin–eosin (HE) staining and special staining for connective tissue: Mallory trichrome, Masson’s trichrome, Verhoeff–Van Gieson and seven-reagent kit for staining reticulin fibres (Reticulin Kit, BioGnost Ltd., Zagreb, Croatia).

## 3. Results

### 3.1. Macroscopic Findings

Gross analysis revealed that 15 out of 136 livers were completely negative. However, of the total of 600 livers (from all red deer shot in Bjelovar–Bilogora County) in one year, only 136 were transported to the Faculty of Veterinary Medicine, resulting in an inaccurate prevalence result, which was therefore omitted from the text. Obtained results are presented in Table 1.

**Figure 1:**
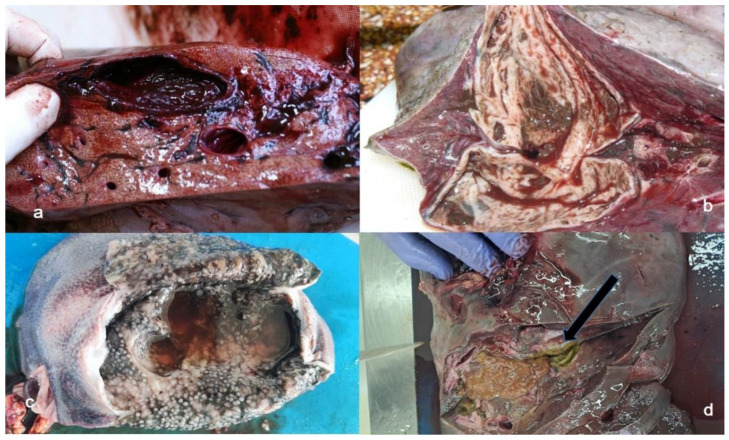
Different types of lesions caused by F. magna infection in red deer: (**a**) migratory channels and pseudocyst; (**b**) pseudocyst; (**c**) large pseudocyst; (**d**) degrading pseudocyst with necrotic content (arrow).

**Figure 2:**
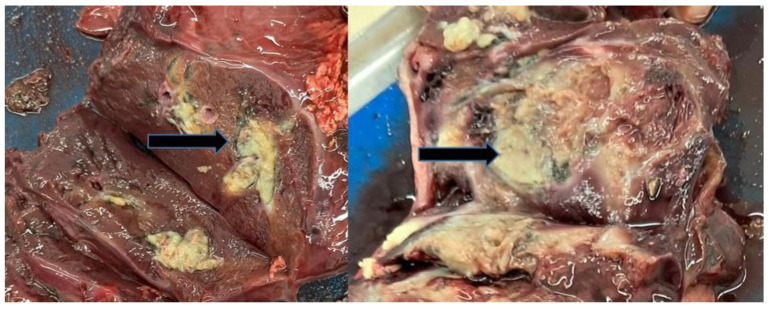
Gross appearance of the regenerative nodule in two red deer livers (arrows).

Data for each locality are presented as mean values. On average, 1.39 juvenile flukes in the migratory phase were detected per infected liver (Figure 1a). The average number of pseudocysts per collected positive red deer liver was 6.62 (range 1 to 45). Some pseudocysts were small (<4 cm diameter) (Figure 1b), while others were as large as a human fist (Figure 1c). The average number of adult flukes per infected liver was 13.27 (ranged from two to 70). Regarding infection intensity, 47.79% of positive livers contained up to six adult flukes, while 30.88% had only juvenile ones. Degrading pseudocysts (Figure 1d) were either absent or present to a maximum of 120 per liver, with an average 7.06 per liver. Occasionally, remnants of the fluke were present in the degrading pseudocysts. Some livers had multifocal to confluent nodules bulging from the liver parenchyma, which were up to 7 cm in diameter (Figure 2).

### 3.2. Microscopic Findings

#### 3.2.1. Migratory Channels

**Figure 3:**
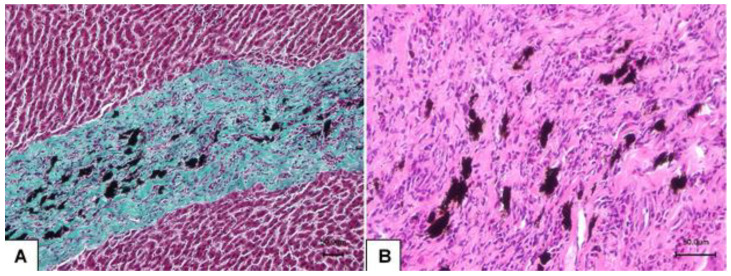
Migration area within the liver parenchyma: (**A**) connective tissue (stained green) containing black pigment iron porphyrin, Masson’s trichrome; (**B**) inflammatory cell infiltrate within the connective tissue, HE.

In the migration area (Figure 3A), an increased amount of connective tissue was present. Accumulations of black iron porphyrin pigment and an inflammatory cell infiltrate, mostly composed of eosinophils, lymphocytes, and plasma cells were visible around the migratory channels (Figure 3B).

#### 3.2.2. Pseudocyst Area

Mallory trichrome staining of pseudocysts showed blue-stained collagen fibres and red-stained muscle cells. Verhoeff–Van Gieson staining highlighted dark blue elastic fibres, mainly located in the direction from the middle to the outer part of the pseudocyst wall, around blood vessels and bile ducts. Reticulin Kit staining demonstrated black-stained reticular fibres dominating the inner part of the pseudocyst wall, between muscle cells, around blood vessels and bile ducts. The pseudocyst wall is formed of collagen, elastic, and reticular fibres, with the presence of muscle cells in some of the analysed pseudocysts. Reticular fibres were mainly located in the inner part of the pseudocyst wall, while elastic fibres were predominantly present from the middle to the outer part of the wall. The inflammatory infiltrate in infected red deer livers predominantly contained lymphocytes, neutrophils and eosinophils. Thickening of blood vessel media and dilatation of sinusoids were observed around pseudocysts. Within pseudocysts, the most common finding included necrotic debris, fluke eggs, and pigmented granules.

#### 3.2.3. Regenerative Area

**Figure 4:**
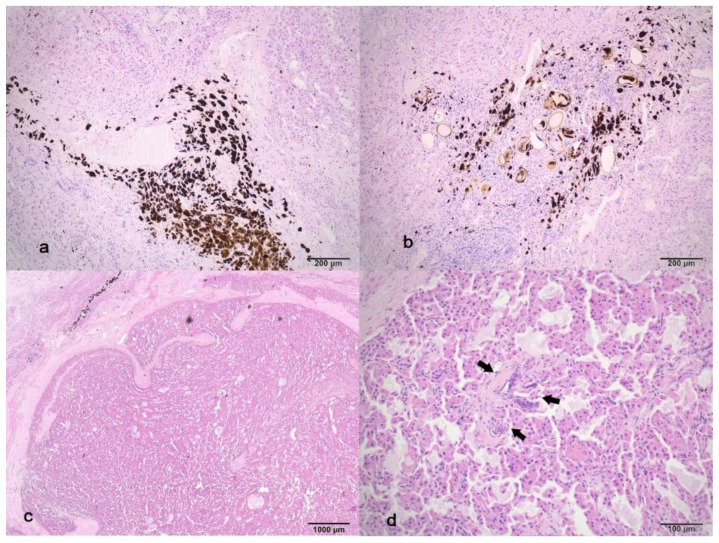
(**a**) Bands of fibrous connective tissue with accumulations of dark brown to black iron porphyrin fluke pigment; separating liver parenchyma in irregular lobules, HEx10; (**b**) bands of fibrous connective tissue with accumulations of dark brown to black iron porphyrin fluke pigment and trematode eggs with a yellow shell, and eosinophilic flocculent material and pigment within the lumen, HEx10; (**c**) a regenerative nodule bounded with fibrous tissue and containing disorganised hyperplastic hepatocytes arranged in irregular trabeculae, HEx2; (**d**) a regenerative nodule at higher magnification, showing disorganised hypertrophic hepatocytes and a structure resembling a portal tract (arrows). Spaces filled with pale eosinophilic fluid between hepatocytes present freezing artefacts, HEx20.

Histologically, the regenerative area showed a disrupted parenchyma with wide and narrow bands of fibrous connective tissue that have divided the parenchyma into pseudolobules of varying size and shape (Figure 4a). These connective tissue septa frequently contained moderate amounts of dark brown to black, anisotropic, granular pigment (iron-porphyrin), along with remnants of elliptical, operculated trematode eggs (Figure 4b). The egg shells were yellowish-brown and were either empty or contained eosinophilic flocculent material mixed with pigment. Additionally, multifocal nodular structures corresponding to regenerative nodules were observed. These nodules were bound with fibrous tissue and consisted of disorganised hyperplastic hepatocytes arranged in irregular trabeculae (Figure 4c). Within the nodules, a single structure resembling a portal tract was rarely apparent (Figure 4d). The nodules were supported by fibrous bands, which occasionally contained blood vessels.

## 4. Discussion

Red deer fascioloidosis is an important parasitic disease of domestic and wild ruminants. Unlike the indigenous European liver fluke *Fasciola hepatica*, *Fascioloides magna* migrates through the liver parenchyma for some time in order to stop migration at a certain point, thus allowing the host to create a pseudocyst. Pseudocysts are formed within the liver parenchyma, not in the bile duct channels. Observed gross lesions include migratory channels caused by juvenile flukes, surrounded by cells of the immune system, mainly eosinophils. During this migratory phase, the juvenile fluke matures while evading the defensive mechanisms of the host. In the case of definitive (red and fallow deer) and dead-end hosts (wild boar, *Sus scrofa*), and more recently also of a roe deer (as an aberrant host), the fluke will eventually end its migratory phase and the organism will create pseudocysts [1,3,10,11,12]. This barrier allows the fluke to live and reproduce, and, in the case of definitive and sometimes even aberrant hosts, to shed eggs into the environment [1,13], thereby contributing to the spread and maintenance of fascioloidosis in nature. Furthermore, the pseudocyst is a prerequisite for the host to survive the infection, as it prevents permanent migration of the juvenile fluke and consequent excessive damage of the liver parenchyma. In established foci of infection, typical hosts usually show no clinical signs of infection. However, potential effects on reproduction efficiency and trophy development in typical hosts are still understudied [14].

In the case of aberrant hosts, mainly mouflon (*Ovis musimon*) and roe deer, *F. magna* infection can have devastating effects on local populations. Therefore, some countries, particularly those along the Danube River, have launched disease control programmes [4,5,7,8]. Different treatment strategies have been applied in different countries, including different drugs, drug carriers, and times of drug exposure (from single dose baits to six-day exposure). Regardless of strategy, all countries reported that full efficacy of the applied treatment was difficult to achieve in environmental conditions, since the majority of studies analysing drug efficacy was previously performed on naturally infected animals that were then kept and treated in enclosures. In such controlled environment, Pybus et al. [15] achieved 90% efficacy of triclabendazole against *F. magna* infection in captive wapiti (*Cervus elaphus canadensis*) following direct drench application of the drug. However, in open habitats, it is impossible to achieve such conditions and treatment efficacy for various reasons, including the following: environmental characteristics and inadequate time of treatment (which is usually in February at the end of the hunting season when natural food is already available), drug withdrawal period, herd hierarchy, deer habits, and reluctance of red deer to consume medicated feed due to its smell and taste [16]. Another factor is the constant decrease in the commercially available drug concentration (from 10% to 5%, as in the case of Tribix^®^), which requires a larger amount of drug applied to corn. This results in increased amounts of drug needed for successful treatment and by that, increased smell and taste of the medicated feed. These factors result in reduced efficacy of the treatment of free-ranging populations.

**Figure 5:**
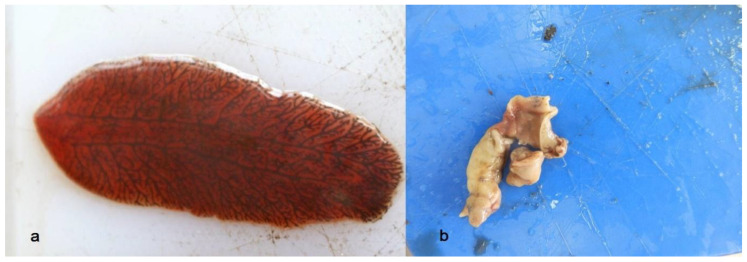
Vital *F. magna* fluke (**a**) and one dead due to the albendazole treatment (**b**).

If the animal consumes the medicated feed, affected pseudocysts contain a variable amount of degraded material and occasionally greyish-yellow remnants of flukes (Figure 5b).

In cases of more numerous or larger pseudocysts, the amount of degraded material can be substantial. If the animal survives the hunting season, such quantities can become potentially toxic. Sometimes, analysis of the livers revealed traces of black pigment and well-developed connective tissue (scars) on the cut surfaces, indicating resorption of the pseudocyst content and tissue reparation. However, there are no reports in the literature on reparation phases in the period between destruction of the pseudocyst and healing, and especially on the formation of new hepatocytes. The previous literature reports severe fibrosis related to *Fasciola hepatica* and *Dicrocoelium dendriticum* infections [17]. However, in these cases, lesions were primarily focused on the formation of connective tissue and cirrhosis. In the case of *F. magna* infection, lesions are more severe and result in the formation of cavities (lumen of former pseudocysts) and a large amount of degraded material. Issa et al. [18] and Bhushan et al. [19] reported that the high regeneration potential of the liver can be compromised by both severe acute liver injury and chronic injury, with aberrant liver architecture and marked fibrosis or even fatty dystrophy of the liver. The first two factors are present in fascioloidosis, while fatty liver is regularly and normally present in red deer stags prior to the rutting season.

## 5. Conclusions

The present study shows that red deer liver possesses not only a high potential of absorption of the degraded pseudocyst material, but also the creation of new hepatic nodules containing fibrous bands, blood vessels and new hepatocytes. With time, part of this area will become new hepatic tissue, while the remainder will form a scar. This type of liver healing could perhaps be better termed as reparation, since in the original translation, the term regeneration represents complete healing with the formation of identical tissue structure to the previous one, while reparation is less perfect and results in partial reconstruction of the previous structure and usually with the formation of scar tissue. In the future, this type of reconstruction of severely injured (acute or chronic) liver tissue, especially in the case of fatty liver, could be used as a model for studying abnormal regenerative responses of the liver.

## Figures and Tables

**Table 1:** Average data (per location and in total) on different gross lesions of the liver in relation to sampling locality.

Hunting Office	N (Livers)	Migratory Fluke	Adult Fluke	Pseudocyst	Degrading Pseudocyst
Bjelovar	15	1.67	13	6.5	5.08
Čazma	38	1.66	18.31	9.11	15.26
Daruvar	21	0.61	8.44	4.22	3.72
Garešnica	22	1.48	9.24	4.62	5.76
Grubišno Polje	13	1.92	10.17	5.08	7.5
Unknown	27	1	19.57	9.78	4.09
Mean		1.39	13.27	6.62	7.06
Min		0.61	8.44	4.22	3.72
Max.		1.92	19.57	9.78	15.26
SD		0.4473	4.0559	2.0189	3.6622
IQR		0.67	9.07	4.49	3.9
95% CI		0.875–1.904	8.106–18.137	4.054–9.048	2.378–11.424

## Data Availability

Requests to access these data should be sent to the corresponding author. No form of artificial intelligence was used for any aspect of drafting this manuscript.
